# Scaling up artificial intelligence to curb infectious diseases in Africa

**DOI:** 10.3389/fdgth.2022.1030427

**Published:** 2022-10-21

**Authors:** Idemudia Otaigbe

**Affiliations:** Department of Medical Microbiology, School of Basic Clinical Sciences, Benjamin Carson (Snr) College of Health and Medical Sciences, Babcock University, Ilishan Remo, Ogun State, Nigeria

**Keywords:** artificial intelligence, infectious diseases, Africa, opportunities, political will, governments, scaling up

## Introduction

Globally, Africa has the highest burden of infectious diseases (1). Furthermore, of the estimated 10 million deaths per year, resulting from infectious diseases, the majority occur in Africa (1). In addition infectious diseases exert adverse clinical and economic impacts on the continent (1). Annually, infectious diseases account for over 227 million years of health life lost and produce an annual productivity loss of over $800 billion (2). In addition, the SARS-CoV-2 pandemic also disrupted Africa's fragile efforts to curb infectious diseases such as Tuberculosis, Malaria and HIV/AIDS. For example, the SARS-CoV-2 pandemic has reversed years of global progress in tackling TB. According to modeling analysis, the COVID-19 pandemic will likely cause an additional 6.3 million cases of TB and 1.4 million TB deaths between 2020 and 2025 (3). Despite the gloomy scenario, wrought by infectious diseases on the continent, a new vista of opportunities has also emerged. For example, the success achieved by Artificial intelligence (AI) platforms in the COVID-19 pandemic (e.g., rapid collection and real-time dissemination of data and vaccine development) can be adapted to combat infectious diseases on the continent (4).

Artificial intelligence (AI) is described as “a machine-based system that can, for a given set of human-defined objectives, make predictions, recommendations, or decisions influencing real or virtual environments. AI systems are designed to operate with varying levels of autonomy” (5). Furthermore AI-based systems may be purely software-based (e.g., voice assistants, image analysis software, search engines, speech and face recognition systems) or embedded in hardware devices such as advanced robots, autonomous cars or drones (6).” Artificial intelligence (AI), offers enormous opportunities to improve patient management and reduce healthcare costs in Africa (7). It also holds immense public health benefits such as drug and vaccine development, disease surveillance, outbreak response and health systems management (7). Africa's health care system stands to benefit from these opportunities (8). For example in Africa, AI could close current gaps in healthcare delivery by extending health care services to rural, underserved populations, improve patient management and disease surveillance (8). In this regard the African Union has launched a continental strategy for Artificial Intelligence (AI) in Africa (9). The continental strategy would enable African countries to develop regulatory frameworks to address AI-related challenges and opportunities (9). Furthermore, the developmental process of the continental strategy would involve integrating existing National AI strategies (9). A National Artificial Intelligence Strategy (NAS) is “a document, ordinarily developed by a government, which sets out its broad, strategic approach to artificial intelligence (AI), including specific areas of focus and activities they will undertake which relate to AI” (10). This definition clearly spells out the role of a National AI strategy. In addition the definition includes the need for the NAS to focus on “specific areas”. In the context of this paper, specific areas of focus would be: (1) harnessing AI-related opportunities to curb infectious diseases and; (2) developing regulatory frameworks to guide the deployment of AI in combatting infectious diseases in Africa. However, several African countries are yet to develop National Artificial Intelligence Strategies (11). In addition, the governance of AI on the African continent is characterized by a diverse spectrum of policy instruments (11). For example, a survey done in 2021 showed that 13 African countries have launched national AI strategies. The same survey also showed that: 6 African countries reported enacting legislation to address challenges associated with AI; 12 have established Centers of Excellence on AI and 3 countries reported issuing ethical guidelines for AI (11). In spite of Africa's slowness to adopt AI, there are massive opportunities for its deployment in Africa's health sector (12). It is therefore necessary to encourage African governments to expedite efforts to include AI in healthcare delivery. However the deployment of AI on the continent is not the sole responsibility of African government as the private sector also has a crucial role to play. In the discussion below are some emerging opportunities which AI presents for the diagnosis, control, prevention and management of infectious diseases in Africa. In addition, challenges regarding the deployment of AI on the continent are mentioned. Finally some recommendations to surmount these challenges are briefly discussed.

## Discussion

### Emerging opportunities

Artificial intelligence can improve the quality of clinical decision-making for the management of infectious diseases. For example, Clinical Decision Support Systems (CDSS) such as Sepsis Watch, are low hanging fruits which can be adopted at institutional levels in Africa (13, 14). Such systems can help to reduce the morbidity and mortality associated with infectious diseases. Sepsis Watch is a Clinical Decision Support System designed for early identification of patients at risk for Sepsis (14). The deployment of such a system in Africa can potentially expedite clinical decision-making regarding sepsis and reduce morbidity and mortality. Furthermore studies have shown the potential of AI to help in diagnosing viral upper respiratory tract infections and thus obviate the need for antibiotics (15). In addition AI has been employed in blocking disease transmission by early detection of cases (16, 17) and the radiological diagnosis of pulmonary tuberculosis (18, 19).

Another emerging opportunity for AI in Africa is precision medicine (20, 21). Precision medicine is “an emerging approach for disease treatment and prevention that takes into account individual variability in genes, environment, and lifestyle for each person” (22). The goal of precision medicine is to ensure the right patient gets the right treatments at the right time (22). In Africa AI can provide precision medicine for diagnosis and treatment of infectious diseases. For example nuclear magnetic resonance (NMR)-based hemozoin detection of malaria is a rapid diagnostic technique which has the potential to detect parasite drug resistance acquisition (20). Another example regards tuberculosis in which AI could be deployed to rapidly detect drug resistance, determine host immunity, individualize drug therapy and predict relapse free cure (21).

Artificial intelligence can also support efforts to curb inappropriate antibiotic use and antimicrobial resistance in Africa (23, 24). The inappropriate use of antibiotics is associated with the emergence of antibiotic resistance, prolonged hospital stay, increased mortality and increased healthcare costs (19). In particular, antibiotic resistance is a global security threat which results in an estimated 700,000 deaths per year globally (25). In addition, a report by the World Health Organization stated that 45% of deaths in Africa and South-East Asia were due to multi-drug resistant bacteria and that extended spectrum beta-lactamase producing *Klebsiella pneumoniae* were associated with elevated deaths in Africa, the Eastern Mediterranean region, South East Asia and the Western Pacific region (26). However, AI-guided antibiotic prescribing could improve the appropriate use of antibiotics and help to curb antibiotic resistance (23). For example, mobile apps could assist physicians in prescribing antibiotics appropriately (24).

Furthermore, the twin problems of inappropriate antibiotic use and antimicrobial resistance are exacerbated by poor adherence to infection prevention and control protocols in Africa (27). Sadly, poor adherence to infection prevention and control protocols drives healthcare associated infections (HAI), including HAI due to multi-drug resistant organisms (27). Health care-associated infections are associated with significant morbidity and mortality. They also exert negative economic impacts on health systems globally (28). On average HAI affect 15% of patients in low- and middle-income countries (LMICs), with attributable mortality estimated at 10% (29, 30). However, a large proportion of HAI are preventable when effective infection prevention and control (IPC) protocols are in place (28). AI offers opportunities to enhance infection prevention and control in healthcare facilities in Africa. For example, hand hygiene practices could be improved through mobile apps or wearable devices that give health workers, visual or audible reminders, regarding hand hygiene (31). In addition AI could be used to analyze routine microbiology laboratory results to detect outbreaks of infections due to multidrug-resistant organisms (32).

Again, Africa's diagnostic microbiology capacity can be enhanced by Artificial Intelligence. In Africa the laboratory diagnosis of infectious diseases still relies on obsolete methods which can miss occult and/or emerging pathogens (33). However the application of AI in diagnostic microbiology has the ability to improve the quality of laboratory processes, increase pathogen detection rates, reduce turn-around time and improve clinical decision making (34). For example AI could help in analyzing images such as Gram stains, ova and parasite analysis and reading bacterial culture plates (34, 35).

Artificial intelligence also has the ability to facilitate drug and vaccine development (36). This has been shown in the COVID 19 pandemic (37). In addition AI can facilitate drug design (e.g., designing drug molecules and predicting drug protein interactions), identifying therapeutic targets and predicting toxicity (38).

Furthermore, the combination of genomics and bioinformatics in the COVID 19 pandemic led to the rapid generation of real time data resulting in expedited public health decision making (39). In addition AI has also proven useful in surveillance and control of Ebola viral haemorrhagic fever (40), malaria (41), tuberculosis (42), and HIV/AIDS (43).

The inclusion of AI in research can also provide insights that could result in a greater understanding of the social determinants of health (44). In addition deploying AI in research could result in the discovery of novel treatments and also aid in mapping the underlying mechanisms, markers, and progression of diseases (44). AI can also help in improving the design and conduct of clinical trials by helping in patient selection and recruitment (45).

### Challenges with deploying artificial intelligence in Africa

AI, offers enormous possibilities to combat infectious diseases in Africa. However, there are significant challenges regarding its deployment on the continent (12). Examples of these challenges include a dearth of technical expertise (12), infrastructural deficits (such as epileptic power supply and poor internet access) and the high costs of deploying AI (12). In addition there are also ethical issues involved in the deployment of AI and a dearth of regulatory frameworks (or legislation) to address these ethical issues (8, 11). Examples of these ethical issues include data privacy i.e., ensuring that sensitive medical information provided by patients on AI platforms, is kept confidential (8, 46, 47); sharing of patients' data without obtaining informed consent (8, 46, 47); inclusiveness and diversity i.e., AI platforms developed in high income nations may not be applicable to the linguistic and socio-cultural diversity of Africa (48); and accountability i.e., who takes responsibility for AI associated errors (8). Finally the deployment of AI in Africa usually involves short term pilot schemes (49). These pilot schemes usually operate as silos and are not integrated into existing health systems (49). Subsequently, they add little value to the quality of healthcare delivery in the nation (49).

### Recommendations

While the above challenges appear large they are however not insurmountable. A combination of political will, effective legislation and adequate funding can surmount these difficulties (12, 50). However, the most important variable is the political will of African governments to adopt AI in healthcare delivery. The term “Political will” is defined as “the commitment of political leaders and bureaucrats to undertake actions to achieve a set of objectives and to sustain the costs of those actions over time” (51). Political will creates an enabling environment for investment in AI and improvement in infrastructure (12). In addition political will provides the opportunity to create regulatory frameworks which will ensure AI is deployed in an equitable, inclusive and sustainable manner that strengthens the health system and provides quality healthcare to the populace (8). Improving political will can however be a complex issue (52). Suggested measures to build political will include: lobbying; building collaborations or strengthening familiarity and trust between governments and individuals (or non-governmental organizations) who are keenly interested in deploying AI to combat infectious diseases in Africa (52). Furthermore it is advisable to include AI into the curricula of undergraduate, specialist and continuing medical education in Africa (53). This would allow the health workforce to acquire the knowledge, attitude and skills required to effectively use AI in healthcare delivery. Similarly African governments should promote the growth of AI education (e.g., undergraduate and postgraduate programs in Artificial intelligence, bioinformatics, etc.) and local tech hubs. These tech hubs can collaborate with the health sector to develop AI Platforms which are sensitive to Africa's socio-cultural diversity. In addition, efforts to scale up AI should not be left to government alone. The private sector should also be involved. For example, private financial institutions can fund the above-mentioned local tech hubs and also provide financing for the deployment of AI platforms in the health sector. Similarly, the private sector can fill infrastructural gaps by investing in power, internet access and other technologies that can support the deployment of AI on the continent (54).

## Conclusion

Artificial intelligence can make significant contributions to Africa's battle against infectious diseases. However, African governments should exhibit the necessary political will required for the successful deployment of Artificial Intelligence on the continent. Furthermore, the private sector should be involved in efforts to deploy Artificial Intelligence in Africa.

## Author contribution

IO conceptualized, drafted, edited and approved the final copy of the manuscript for submission.

## Conflict of interest

The author declares that the research was conducted in the absence of any commercial or financial relationships that could be construed as a potential conflict of interest.
